# Comparative metabolomics reveal key pathways associated with the synergistic activities of aztreonam and clavulanate combination against multidrug-resistant *Escherichia coli*

**DOI:** 10.1128/msystems.00758-23

**Published:** 2023-10-13

**Authors:** Jiayuan Zhang, Hai Yang, Lei Zhang, Zhihua Lv, Mingming Yu, Sherwin K. B. Sy, Yuanchao Zhan

**Affiliations:** 1 Ocean University of China, Qingdao, China; 2 Department of Pharmacy, Affiliated Qingdao Central Hospital of Qingdao University, Qingdao Cancer Hospital, Qingdao, China; 3 Department of Laboratory Medicine, Affiliated Qingdao Central Hospital of Qingdao University, Qingdao, China; 4 Laboratory for Marine Drugs and Bioproducts of Qingdao National Laboratory for Marine Science and Technology, Qingdao, China; 5 Department of Statistics, State University of Maringá, Maringá, Paraná, Brazil; Dalhousie University, Halifax, Nova Scotia, Canada

**Keywords:** metabolomics, *Escherichia coli*, clavulanate, aztreonam, synergy

## Abstract

The use of combination antibiotics has been useful in the treatment of multidrug-resistant bacterial infections. The synergistic effect of amoxicillin/clavulanate and aztreonam combination against *Escherichia coli* carrying New Delhi metallo-β-lactamase (NDM) was primarily due to clavulanate inhibiting aztreonam degradation. In the present study, we employed metabolomic analysis to investigate the downstream changes in *E. coli* after treatment with aztreonam and clavulanate. *E. coli* metabolomes were compared at 1 and 24 h following treatments with clavulanate (4 µg/mL) and aztreonam (4 µg/mL) alone and in combination. Excluding false positives, 198 metabolites were identified to be affected by antibiotic treatment. Aztreonam/clavulanate combination inhibited cell wall synthesis more aggressively at 1 h and 24 h than aztreonam alone. The purine and pyrimidine metabolism, the central carbon metabolism, and the amino acid metabolism were also disrupted resulting in a prolonged bactericidal effect. This study reveals the synergistic killing mechanism of clavulanate and aztreonam combination against *E. coli* harboring NDM and provides a theoretical basis for the combined use of aztreonam/clavulanate in the treatment of multidrug-resistant *E. coli* infection.

**IMPORTANCE:**

Multidrug-resistant *Escherichia coli* is a major threat to the health care system and is associated with poor outcomes in infected patients. The combined use of antibiotics has become an important treatment method for multidrug-resistant bacteria. However, the mechanism for their synergism has yet to be explored.

## INTRODUCTION

In the last 15 years, intensive care and infectious disease physicians have faced novel challenges in the treatment of severe infections in critically ill patients in intensive care units, due to the selection for and spread of multidrug-resistant Gram-negative bacteria (MDR-GNB) (1, 2). MDR *Escherichia coli*, as the most common MDR-GNB, is a major threat to the health care system and is associated with poor outcomes in infected patients (3). *E. coli* is a common pathogen that causes enteric foodborne illness, urinary tract infections, bloodstream infections, and gastroenteritis diseases in animals and humans worldwide (4, 5). In the face of menacing MDR *E. coli*, the use of multiple antibiotics as polytherapy has become a widely accepted treatment method in clinical practice (6, 7). Clavulanic acid was one of the first β-lactamase inhibitors to be discovered; it was produced by *Streptococcus clavuligerus* where the name clavulanic acid came from (8). The β-lactam ring of clavulanic acid binds irreversibly to bacterial β-lactamase preventing it from inactivating β-lactam antibiotics (9). As the only monobactam on the market, aztreonam impedes the final stage of bacterial membrane synthesis by inhibiting the penicillin binding protein; the β-lactam ring in aztreonam is not fused to another ring (10, 11).

Amoxicillin/clavulanate in combination with aztreonam has been shown to have synergistic activities against MDR *E. coli* in numerous studies (12–14). This combination has a wide range of potential clinical applications because it is cost-effective and well tolerated. We have previously found that aztreonam/clavulanate or aztreonam/amoxicillin/clavulanate had almost the same *in vitro* bactericidal effect against MDR *E. coli* (15), indicating that there is minimal benefit in having amoxicillin present with aztreonam/clavulanate. In the present study, we employed metabolomic analysis to elucidate the time-dependent changes in MDR *E. coli*, when treated with the aztreonam/clavulanate combination. The results provide some molecular basis for the synergistic activities of aztreonam/clavulanate combination against MDR *E. coli*, as characterized by the metabolic changes within the bacteria.

## RESULTS

### *In vitro* antimicrobial susceptibility

**Fig 1:**
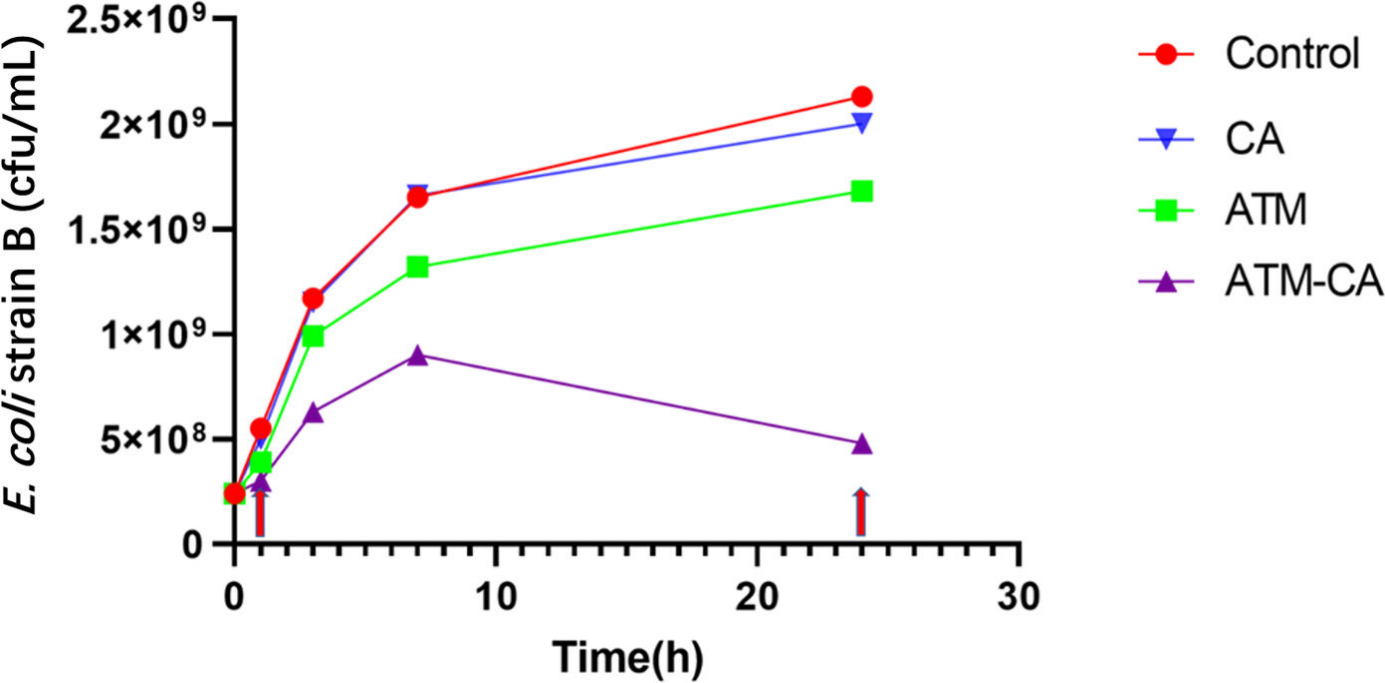
Time-kill curves of *E. coli* strain B after antibiotic treatments: control (control), clavulanic acid (CA), aztreonam (ATM), and aztreonam/clavulanate combination (ATM-CA). Red arrows indicate selected sampling points for metabolomic evaluation.

Table 1 shows the resistance genes and antibiotic susceptibility for three MDR *E. coli* strains. All three strains of *E. coli* contained the New Delhi metallo-β-lactamase (NDM) gene and other resistance genes that rendered the bacteria resistant to aztreonam alone. The minimum inhibitory concentrations (MICs) of the quality control strains, *E. coli* ATCC 25922 and *E. coli* ATCC 35218*,* against aztreonam were both less than 0.5 mg/L, meeting the standards set by the Clinical and Laboratory Standards Institute (CLSI). The combination of clavulanic acid and aztreonam produced significant synergy and reduced the MIC of aztreonam to below its clinical breakpoint (16, 17). The CLSI breakpoints used for the interpretation of aztreonam MIC results were as follows: ≤4 mg/L (susceptible), 8 mg/L (intermediate), and ≥8 mg/L (resistant) for *E. coli* (18). We chose *E. coli* B for the time-kill experiment and metabolomic analysis, since it has the largest fold difference in MIC between aztreonam monotherapy and aztreonam/clavulanate combination. The time-kill curves of the four treatment groups are shown in Fig. 1; the bacterial growth of the clavulanate group is almost the same as that of the control group, indicating that clavulanic acid has limited activity without a partnering β-lactam. The bacterial growth in both the aztreonam and aztreonam/clavulanate groups was inhibited; the inhibition was more pronounced in the combined aztreonam/clavulanate group. After 24 h, the difference between the two groups became even more apparent. Consequently, samples were collected at 1 h and 24 h to investigate the impact of different drug treatments on the metabolites in *E. coli*. No bactericidal effect was observed given that the time-kill experiment was carried out at a high initial inoculum size of 10^8^ cfu/mL to obtain sufficient metabolites for quantification.

**TABLE 1:** Minimum inhibitory concentrations of aztreonam alone, clavulanate alone, and aztreonam/clavulanate combination against *E. coli* clinical isolates, as well as β-lactamase genes encoded in each isolate

Strains	β-Lactamase encoded	MIC (mg/L)			FICI^*d*^
		ATM^*a*^	CA^*b*^	ATM-CA^*c*^	
Control					
*E. coli* ATCC 25922		<0.5	–	–	–
*E. coli* ATCC 35218		<0.5	–	–	–
*E. coli*					
A	NDM-1, CTX-M-55, TEM-1B	32	64	0.25/0.5	0.0156
B	NDM-13, TEM-1B, TEM-141, CTX-M-55, OXA-1	64	32	0.25/0.5	0.0195
C	NDM-9, CTX-M-65, TEM-34, TEM-1B, OXA-1	128	32	4/2	0.0938

^*a*^ ATM, aztreonam.

^*b*^ CA, clavulanic acid.

^*c*^ ATM-CA, aztreonam/clavulanate combination.

^*d*^ FICI, fractional inhibitory concentration index.

### Aztreonam and clavulanic acid alone or in combination caused disruptions in overall metabolites

**Fig 2:**
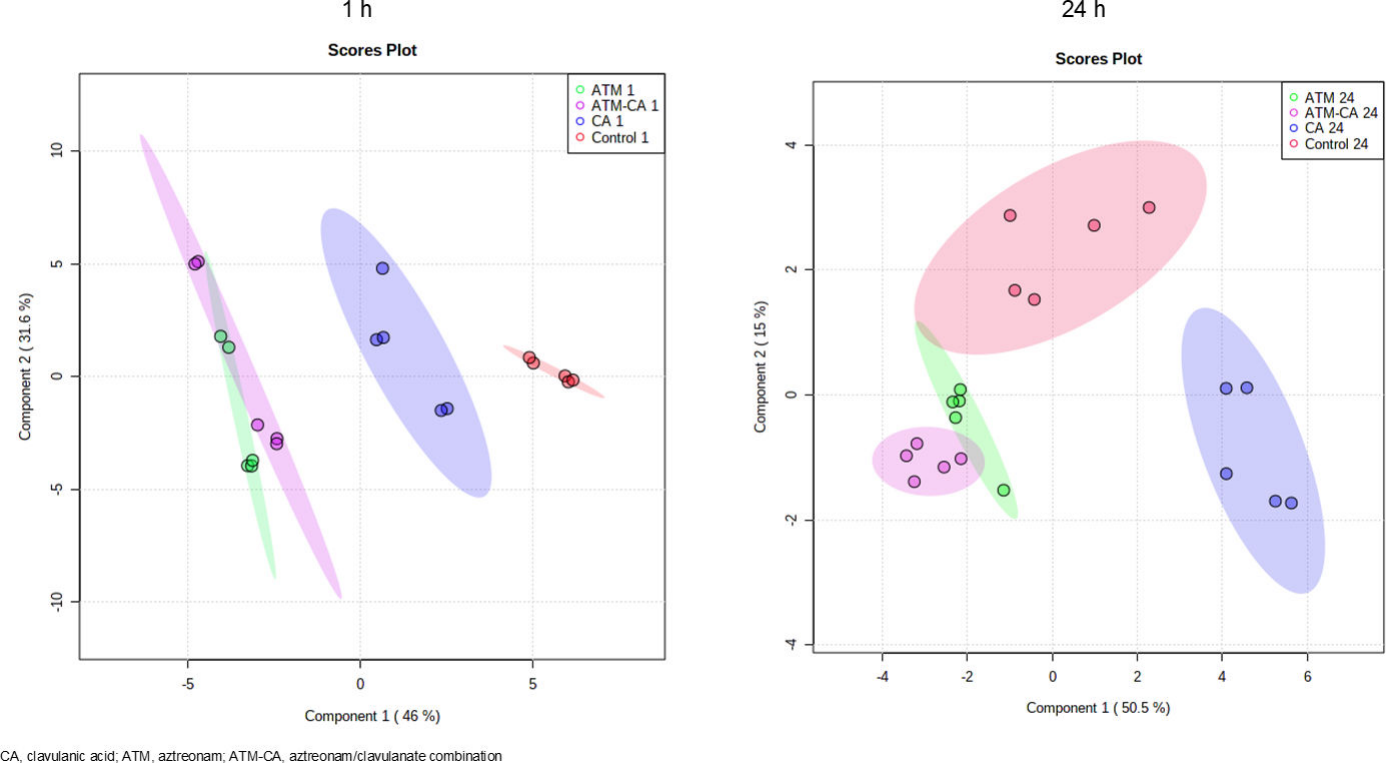
PLS-DA score plots of the first two principal components for *E. coli* strain B metabolite levels in the control group (control), clavulanic acid group (CA), aztreonam group (ATM), and aztreonam/clavulanate combination group (ATM-CA) at 1 and 24 h.

**Fig 3:**
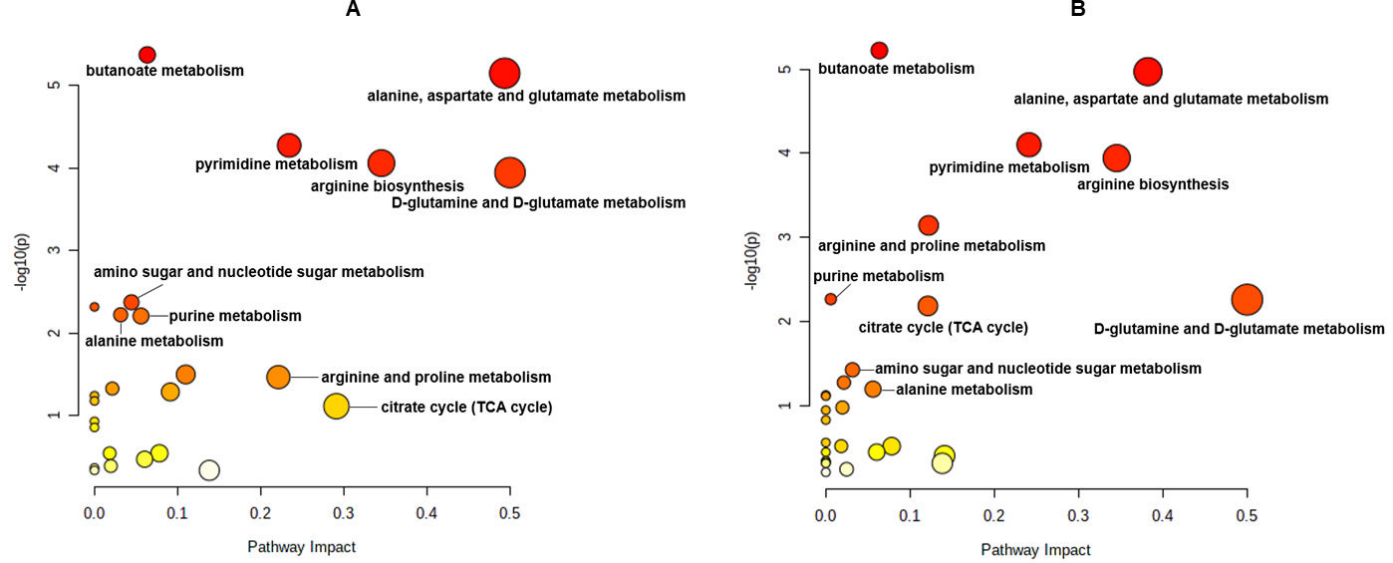
Pathway analysis of significantly affected metabolites following the treatments with clavulanic acid, aztreonam alone, and aztreonam/clavulanate combination (*P* < 0.05 and fold change ≥2) at (**A**) 1 h and (**B**) 24 h. The pathway enrichment analysis was based on Kyoto Encyclopedia of Genes and Genomes database (http://www.genome.jp/kegg/pathway.html). TCA, tricarboxylic acid.

A total of 198 metabolites were identified to be affected by the antibiotic treatments. The relative standard deviation of the quality control samples was less than 20%, and the error rate was within the acceptable standard range for metabolomics. The results of the partial least squares discriminant analysis (PLS-DA) showed that the control group and the treated group have stark differences after 1 h and 24 h post-treatment (Fig. 2). Both the aztreonam and aztreonam/clavulanate groups were separated from the control group after 1 h (component 1, 46%; component 2, 31.6%). However, there was an overlap between these two groups. After 24 h, there was minimal overlap between the combination group and the control group, and the distance between the aztreonam/clavulanate group and the control group was greater compared to the distance between the aztreonam and control groups (component 1, 46%; component 2, 31.6%). A close examination of pathways of the affected metabolites with significant changes indicated that drug treatments mainly affected amino acid metabolism, central carbon metabolism, purine and pyrimidine metabolism, and amino sugar and ribose metabolism (Fig. 3). The specific metabolic changes are summarized in Table 2.

**TABLE 2:** Sequence of metabolomic changes in *E. coli* after aztreonam and clavulanic acid treatments as monotherapy and in combination

Time	ATM-CA	ATM	CA
1 h	Cell wall synthesis	Cell wall synthesis	Cell wall synthesis
	↑GlcNAc; ↑N-acetylmuramate ↑D-glucosamine; ↓UDP-GIcNAc ↑LL-2,6-diaminopimelate	↑GlcNAc; ↓UDP-GIcNAc; ↓N-succinyl-L-diaminopimelate; ↑LL-2,6-diaminopimelate	↑GlcNAc; ↓UDP-GIcNAc; ↑LL-2,6-diaminopimelate
	Purines and pyrimidine	Purines and pyrimidine	Purines and pyrimidine
	↑adenosine; ↑xanthine; ↑allantoin; ↓dihydrouracil; ↓thymine; ↑thymidine; ↑(S)-dihydroorotate; ↑uracil	↑allantoin; ↓thymine; ↑thymidine; ↑(S)-dihydroorotate; ↓dihydrothymine	↑adenosine; ↑allantoin; ↓dihydrouracil; ↓thymine; ↑(S)-dihydroorotate; ↑uracil
	Central carbon metabolism	Central carbon metabolism	Central carbon metabolism
	↓xylulose 5-phosphate; ↓fructose 6-phosphate; ↓2-oxoglutarate; ↓acetyl-CoA; ↓L-malate; ↓succinate	↓xylulose 5-phosphate; ↓fructose 6-phosphate; ↓succinate	↓succinate
24 h	Cell wall synthesis	Cell wall synthesis	Cell wall synthesis
	↑N-acetylmuramate; ↑UDP-GIcNAc; ↑N-succinyl-L-diaminopimelate; ↓LL-2,6-diaminopimelate; ↑2,3,4,5-tetrahydrodipicolinate; ↑L-aspartic 4-semialdehyde	↓N-succinyl-L-diaminopimelate	↑UDP-GIcNAc
	Amino acids	Amino acids	Amino acids
	↑succinic acid semialdehyde; ↓L-glutamine; ↑4-aminobutanoate; ↑L-glutamate; ↓L-2-aminoadipate ↑N-succinyl-L-glutamate 5-semialdehyde; ↑N2-succinyl-L-ornithine; ↓5-guanidino-2-oxopentanoate; ↓N-acetylcitrulline; ↑L-citrulline; ↑cadaverine; ↓5-aminopentanamide;	↑4-aminobutanoate; ↑L-glutamate; ↓N2-succinyl-L-ornithine; ↓L-2-aminoadipate	↑N-succinyl-L-glutamate 5-semialdehyde; ↓5-guanidino-2-oxopentanoate; ↓N-acetylcitrulline; ↑L-citrulline
	Purines and pyrimidine	Purines and pyrimidine	Purines and pyrimidine
	↓xanthine; ↓allantoin; ↓thymine; ↓thymidine; ↓cytidine; ↓(S)-dihydroorotate; ↓dihydrothymine; ↓uracil	↓allantoin; ↓cytidine; ↓uracil	↓allantoin; ↓cytidine; ↓(S)-dihydroorotate; ↓dihydrothymine; ↓uracil
	Central carbon metabolism	Central carbon metabolism	Central carbon metabolism
	↓xylulose 5-phosphate; ↓fructose 6-phosphate; ↓2-oxoglutarate; ↓fumarate; ↓succinate	↓xylulose 5-phosphate; ↓fructose 6-phosphate; ↓succinate	↓succinate

^*a*^ ATM-CA, aztreonam/clavulanate combination.

^*b*^ ATM, aztreonam.

^*c*^ CA, clavulanic acid.

### Effects on peptidoglycan biosynthesis and amino sugar and nucleotide sugar metabolism

**Fig 4:**
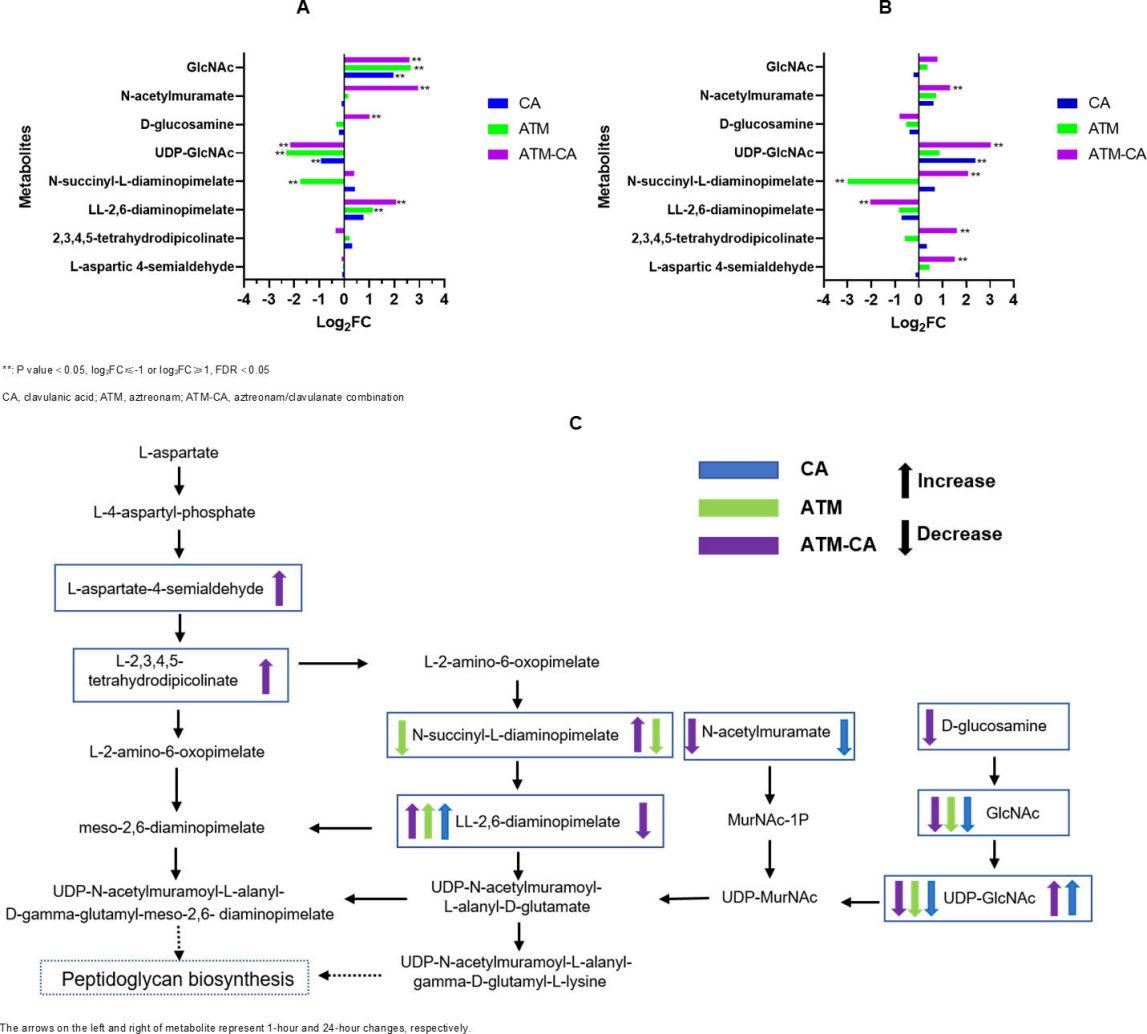
Perturbations of peptidoglycan and amino sugar biosynthesis in *E. coli* treated with antibiotics alone and in combination at 1 h (**A**) and 24 h (**B**); illustration of peptidoglycan and amino sugar biosynthesis pathway affected by treatment of aztreonam and clavulanic acid alone and their combination (**C**).

Aztreonam and clavulanic acid alone or in combination significantly altered the abundance of nine metabolites in the peptidoglycan biosynthesis and amino sugar and nucleotide sugar metabolisms (Fig. 4A and B). In all subsequent comparisons, the *E. coli* strain B control group without drug treatment at the same collection timepoint during the time-kill experiment was used to compare with the same strain treated with antibiotics. At 1 h after the addition of aztreonam/clavulanate, there was a significant increase in the levels of N-acetylglucosamine (GlcNAc), N-acetylmuramate, D-glucosamine, and LL-2,6-diaminopimelate (log_2_ FC = 2.59, 2.95, 1.01, and 2.01; *P*-value = 1.9 × 10^−5^, 1.8 × 10^−7^, 0.024, and 6.3 × 10^−4^, respectively) and a significant decrease in UDP-GlcNAc (log_2_ FC = −2.14; *P*-value = 0.001). The results at 24 h after treatment were markedly different from that of 1 h; there was a significant increase of N-succinyl-L-diaminopimelate, 2,3,4,5-tetrahydrodipicolinate, L-aspartic 4-semialdehyde, UDP-GlcNAc, and N-acetylmuramate (log_2_ FC = 2.08, 1.60, 1.53, 3.04, and 1.33; *P*-value = 2.4 × 10^−6^, 0.001, 2.4 × 10^−5^, 6.4 × 10^−6^, and 0.037, respectively), and a significant decrease of LL-2,6-diaminopimelate (log_2_ FC = −2.03; *P*-value = 0.009). Compared with the aztreonam/clavulanate group, the clavulanate group had far fewer altered metabolites; only the level of GlcNAc (log_2_ FC = 1.95; *P*-value = 0.001) increased after 1 h and UDP-GlcNAc (log_2_ FC = 2.39; *P*-value = 0.02) increased after 24 h. The aztreonam group showed a significant increase in GlcNAc and LL-2,6-diaminopimelate (log_2_ FC = 2.64 and 1.13; *P*-value = 0.002 and 0.006, respectively) and a significant decrease in UDP-GlcNAc and N-succinyl-L-diaminopimelate (log_2_ FC = −2.30 and −1.76; *P*-value = 0.02 and 0.01, respectively) at the 1 h; only N-succinyl-L-diaminopimelate (log_2_ FC = −2.99; *P*-value = 0.007) was still significantly reduced at 24 h. Illustration of affected metabolites in the peptidoglycan and amino sugar biosynthesis pathway is shown in Fig. 4C.

### Effects on the synthesis and metabolism of amino acids

**Fig 5:**
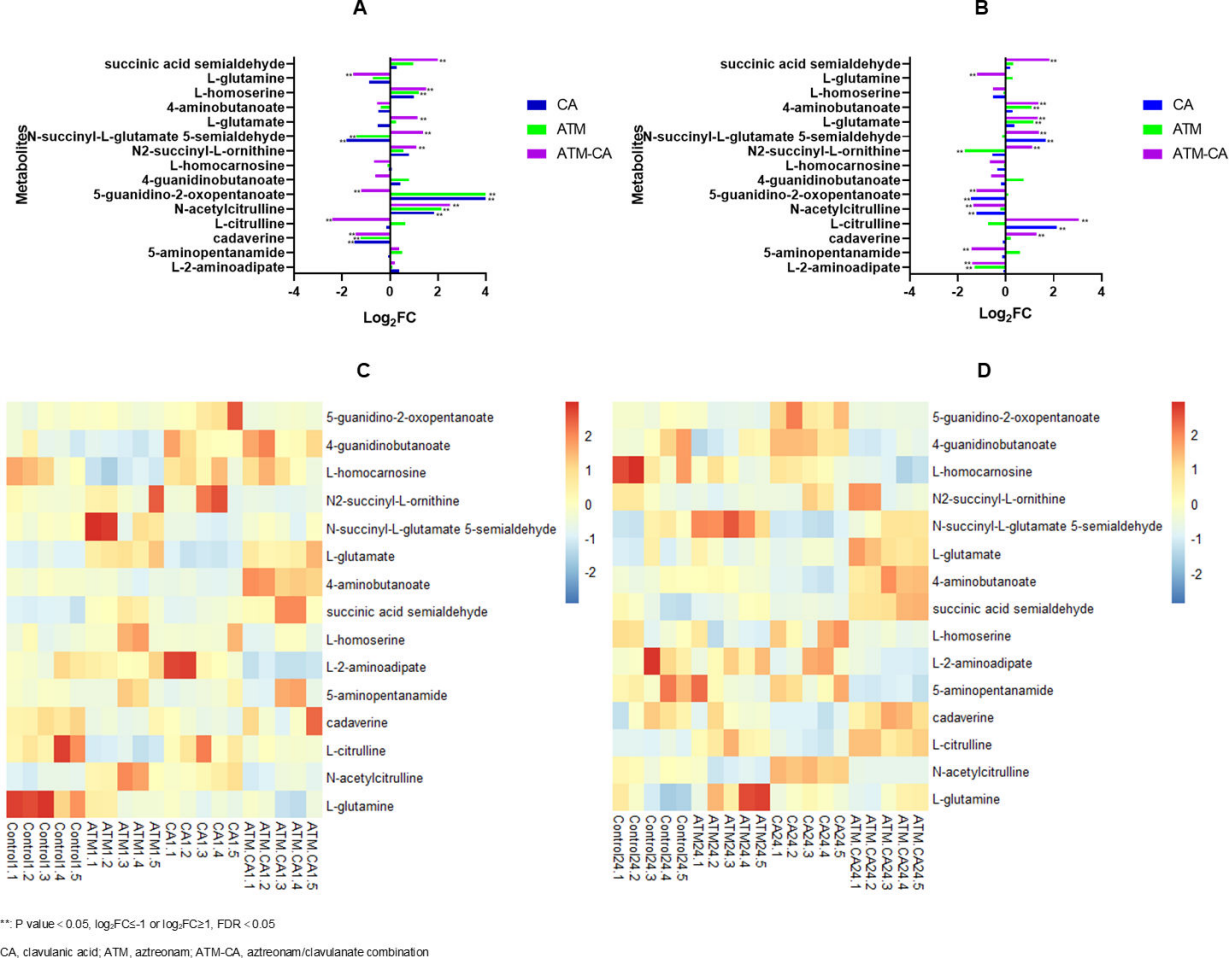
Perturbations of amino acids in *E. coli* treated with antibiotics alone and in combination at 1 h (**A**) and 24 h (**B**). Heatmap shows the normalized relative intensity of the significantly disrupted metabolites involved in amino acid metabolism at 1 h (**C**) and 24 h (**D**) in five replicates per treatment group.

The biosynthesis and metabolism of bacterial amino acids were affected by drug treatments (Fig. 5A and B). L-glutamate and 4-aminobutanoate involved in alanine, aspartate, and glutamate metabolism have varying degrees of increased abundance in both aztreonam (log_2_ FC = 1.16 and 1.09; *P*-value = 0.04 and 0.0002, respectively) and aztreonam/clavulanate treatment groups (log_2_ FC = 1.34 and 1.35; *P*-value = 0.0001 and 1.9 × 10^−5^, respectively) at 24 h after administration. Succinic semialdehyde in the aztreonam/clavulanate-treated group accumulated at both 1 h and 24 h, whereas L-glutamine was significantly reduced. L-2-aminoadipate, 5-aminopentanamide, and cadaverine (log_2_ FC = −1.40,–1.43, and 1.27; *P*-value = 0.03, 2.3 × 10^−5^, and 0.03, respectively) involved in lysine biosynthesis and metabolism have been altered significantly after 24 h of the combination treatment. Multiple metabolites related to the arginine and proline metabolic pathways (DL-citrulline, N-acetylcitrulline, 5-guanidino-2-oxopentanoate, 4-guanidinobutanoate, L-homocarnosine, N2-succinyl-L-ornithine, and N-succinyl-L-glutamate 5-semialdehyde) have been affected; their levels were different compared to the control group. The changes in amino acid metabolisms were extensive and complex, with the aztreonam/clavulanate group apparently interfering the most among the treatment groups (Fig. 5C and D).

### Effect on the synthesis and metabolism of purines and pyrimidines

**Fig 6:**
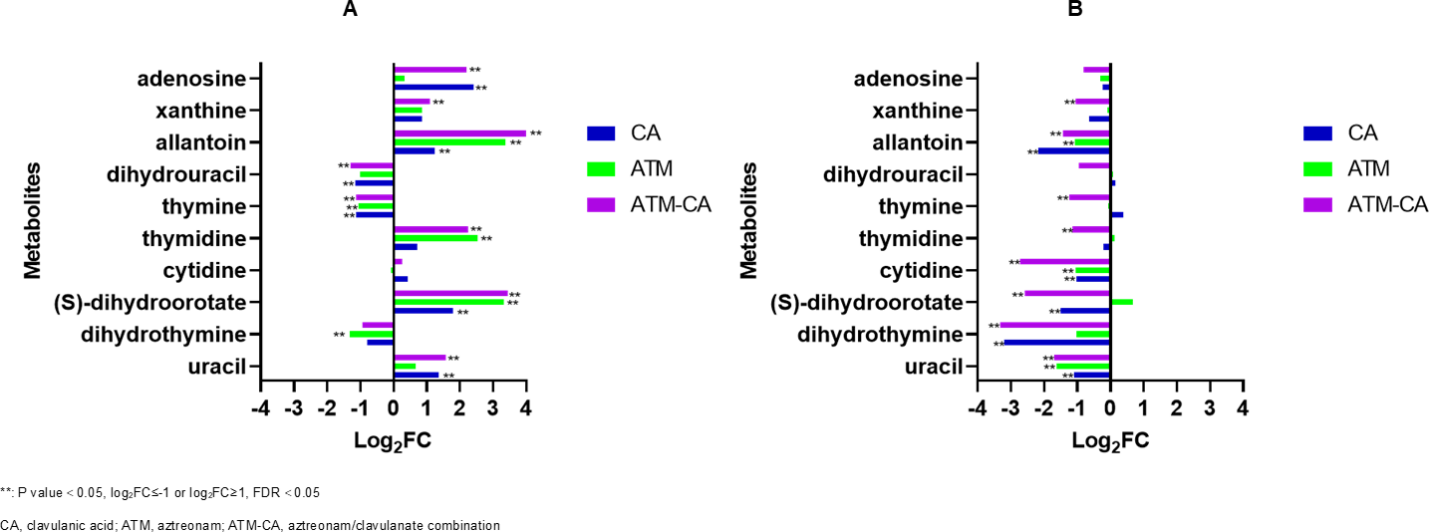
Perturbations of purines and pyrimidine biosynthesis in *E. coli* treated with antibiotics alone and in combination at 1 h (**A**) and 24 h (**B**).

After 1 h of drug administration, the three treatment groups showed remarkable consistency in terms of purine and pyrimidine metabolisms. The levels of thymine, dihydrouracil, and dihydrothymine were decreased, whereas the abundance of other metabolites increased in varying degrees in the three groups at 1 h after administration (Fig. 6A). At 24 h after administration, metabolites such as uracil, dihydrothymine, (S)-dihydroorotate, cytidine, thymidine, thymine, cytidine, allantoin, and xanthine (log_2_ FC = −1.1 to −3.3) have decreased significantly after aztreonam/clavulanate treatment, while only uracil (log_2_ FC = −1.63; *P*-value = 0.04) in the aztreonam group has decreased (Fig. 6B). However, purine metabolism in the aztreonam group returned to normal at 24 h post-treatment.

### Interference with central carbon metabolism

**Fig 7:**
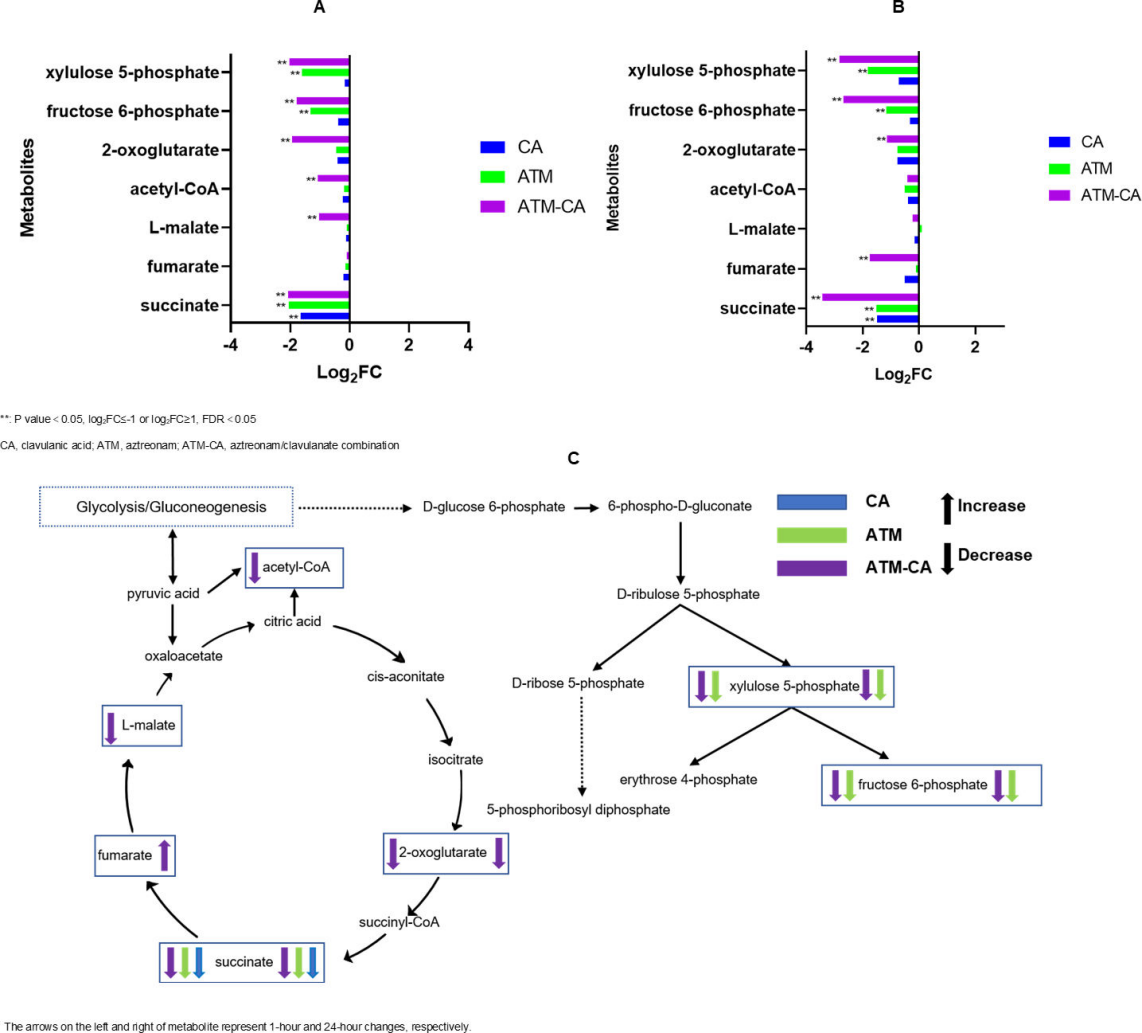
Perturbations of central carbon metabolism in *E. coli* treated with antibiotics alone and in combination at 1 h (**A**) and 24 h (**B**); illustration of central carbon metabolism pathway affected by treatment of aztreonam and clavulanic acid alone and their combination (**C**).

Even though β-lactam’s mechanism of action is to disrupt cell wall synthesis, these antibiotics also interfered with the central carbon metabolism pathway, which functions to provide energy for the bacteria (Fig. 7A and B). Disruptions in the tricarboxylic acid (TCA) cycle and the pentose phosphate pathway are illustrated in Fig. 7C. In the TCA cycle, the most significant changes were associated with the aztreonam/clavulanate group. The levels of 2-oxoglutarate, acetyl-CoA, L-malate, and succinate (log_2_ FC = -1.0 to −2.1) at 1 h and 2-oxoglutarate, fumarate, and succinate (log_2_ FC = −1.1, –1.7, and −3.4; *P*-value = 0.04, 0.0003, and 1.2 × 10^−5^, respectively) at 24 h after administration were remarkably reduced. After treatments with either clavulanate or aztreonam alone, only the level of succinate showed significant downregulation. In the pentose phosphate pathway, we found that fructose 6-phosphate and xylulose 5-phosphate were significantly reduced in both aztreonam and aztreonam/clavulanate groups at 1 and 24 h, but the decline was greater in the aztreonam/clavulanate group.

## DISCUSSION

*E. coli* is one of the most common Gram-negative pathogens in the clinic and is accompanied by a growing problem of antibiotic resistance, which poses a serious threat to human health (19). Aztreonam combined with clavulanate has been shown to exert a synergistic effect on *E. coli* that carries the NDM resistance gene (15). The present study applied metabolomic profiling to investigate the mechanisms underlying the synergistic effects of aztreonam combined with clavulanate against *E. coli*. The results show that aztreonam plays an important role in interfering with the growth and reproduction of bacteria for a short period of time, and clavulanate aggravates and prolongs this disruption, suggesting that the β-lactamases in these bacteria quickly counter the bactericidal effect of aztreonam by degrading it (20). In 6 h, >90% of aztreonam is degraded in bacteria harboring ESBLs and NDM (21). The combination of aztreonam and clavulanate inhibits the metabolism of *E. coli* by interfering with metabolic pathways of peptidoglycan and amino acid biosynthesis, central carbon, purine and pyrimidine metabolisms.

The pharmacological effects of aztreonam are due to its high affinity for the penicillin-binding protein 3 on the cell membrane of Gram-negative bacilli, by inhibiting cell wall synthesis and blocking filament formations that mediate bacterial cell division; consequently, bacteria undergo cell lysis and death (22). In this metabolomic study, we first focused on the changes in metabolic pathways during the synthesis of cell walls. Numerous metabolites in the peptidoglycan synthesis pathway were found to be disrupted significantly by aztreonam or aztreonam/clavulanate treatments. L-aspartic 4-semialdehyde, 2,3,4,5-tetrahydrodipicolinate, N-succinyl-L-diaminopimelate, and LL-2,6-diaminopimelate were initially synthesized from L-aspartate to meso-2,6-diaminopimelate and then to peptidoglycan, an important intermediate product of this process. L-aspartic 4-semialdehyde and 2,3,4,5-tetrahydrodipicolinate had significant accumulation at 24 h after treatment of aztreonam/clavulanate but not their monotherapies. The increase in LL-2,6-diaminopimelate was short-lived after 1 h of treatment in all three groups but returned to normal at 24 h. We hypothesized that the accumulation was because these metabolites were not being used by the downstream processes for the production of proteoglycan.

In addition to the effects on cell wall synthesis, the metabolomic results have shown that aztreonam/clavulanate combination significantly reduced purine and pyrimidine metabolisms of *E. coli*. Most metabolites associated with purine and pyrimidine metabolisms showed consistency in both aztreonam- and aztreonam/clavulanate-treated groups; we focused on the more differentiated metabolic changes after aztreonam/clavulanate treatment to explore the effect of adding clavulanate on the antibacterial effects of aztreonam.

Adenosine plays an important role in energy transfer as adenosine triphosphate and adenosine diphosphate. Studies have shown that transient or permanent damage of cell membranes during trauma will lead to rapid formation of adenosine (23); when present in sufficiently high levels, adenosine can act as an immunotoxin and a metabotoxin (24). Compared with aztreonam, aztreonam/clavulanate treatment resulted in a significant increase in bacterial adenosine, possibly due to permanent damage to the cell membrane or inhibition of adenosine kinase (ADK); the appearance of a large amount of adenosine will in turn inhibit the growth and proliferation of bacteria and stimulate the regulatory function of ADK to return adenosine to normal levels (25). Adenosine abundance only had a significant effect at the beginning of antibiotic treatment; the adenosine levels returned to normal levels at 24 h after treatment.

The cell membranes of bacteria in the aztreonam/clavulanate-treated group received greater damage, directly or indirectly affecting the central carbon metabolism pathway. There were significant declines in six metabolites in the aztreonam/clavulanate-treated group involving the central carbon metabolic pathway. The stability of central carbon metabolism directly affects the survival of bacterial cells (26). The change in fumarate and oxoglutarate levels in the citric acid cycle also interferes with the normal redox reaction of nicotinamide adenine dinucleotide (NAD+). NAD+ is both a crucial coenzyme and a co-substrate for various metabolic reactions in all living cells (27, 28). An equilibrium in NAD+ levels is essential for cell energy homeostasis, survival, proliferation, and function (29, 30). Compared with the aztreonam/clavulanate treatment, only succinate was significant inhibited in the citric acid cycle in the aztreonam-treated group, without involving NAD+, whereas aztreonam/clavulanate treatment affected NAD+, as well, which may be another important factor for the aztreonam/clavulanate combination to maintain a longer sterilization effect.

In conclusion, the addition of clavulanic acid can greatly improve the bactericidal effect of aztreonam on MDR *E. coli* that carries drug-resistant genotypes. The results of our metabolomic study revealed that the *in vitro* treatment of *E. coli* with the aztreonam/clavulanate combination inhibited the synthesis of proteoglycan, thus affecting the normal synthesis of cell wall and cell membrane, disrupting the equilibrium of purine and pyrimidine metabolism, central carbon metabolism, and amino acid metabolism, thereby maintaining a prolonged bactericidal effect.

## MATERIALS AND METHODS

### Clinical isolates, antibiotics, and reagents

Three clinical isolates of *E. coli* were collected from sputum of patients with pulmonary infection at the affiliated hospital of Qingdao University. *E. coli* ATCC 25922 and 35215 (Shanghai Sango Biotechnology Co., Ltd. Shanghai, China) were used as quality control strains for susceptibility testing purpose. *E. coli* ATCC 35218, a β-lactamase-producing strain, is recommended as the quality control organism for the β-lactam–β-lactamase inhibitor agents. Next-generation sequencing was used to determine antibiotic resistance genes in each clinical isolate. Analytical-grade clavulanate and aztreonam (Shanghai Macklin Biochemical Co., Ltd., Shanghai, China) solutions were prepared according to CLSI guidelines (18).

### Susceptibility testing

The MICs of aztreonam and clavulanate alone or in combination against three *E. coli* clinical isolates were determined according to CLSI guidelines (18, 31). *E. coli* strains were prepared at a density of 0.5 McFarland and then diluted into each well of the 96-well plate to a final density of 5 × 10^5^ cfu/mL. The plate was pre-treated with antibiotics wherein each well contained twofold increment in drug concentrations. The incubation was carried out for 20 h at 35°C ± 2°C. The analysis was performed in triplicate.

### Time-kill experiment for metabolomic profiling

To maximize the differences in affected metabolites between monotherapy and combination therapy, the clinical isolate exhibiting the greatest fold difference in MIC between aztreonam monotherapy and aztreonam/clavulanate combination was selected to determine its time-kill kinetics; subsequent metabolomic experiments were performed on this isolate from samples collected during the time-kill study. Briefly, *E. coli* culture was prepared on a nutrient agar plate from the frozen stock (−80°C) and incubated for 16 to 18 h at 37°C. A single colony of *E. coli* was inoculated into 15 mL Mueller-Hinton broth (MHB) and incubated at 37°C for an overnight culture. To obtain sufficient cells for metabolomic extraction, bacteria were cultured to logarithmic growth with a starting inoculum size of approximately 10^8^ cfu/mL, examined using 600-nm optical density, before drugs were added (32). Bacterial culture was treated with either 4 µg/mL clavulanate (clavulanate group), 4 µg/mL aztreonam (aztreonam group), or 4 µg/mL aztreonam and 4 µg/mL clavulanate (aztreonam/clavulanate group). The concentration of aztreonam was based on its clinical breakpoint (18). Bacterial culture without any antibiotic served as control sample (control group). Determination of bacterial density was carried out at 1, 4, 8, and 24 h to obtain time-kill curves.

### Preparation of cellular metabolite extracts

Metabolite determinations were carried out from the time-kill experiments. Five biological replicates were prepared for each treatment condition. Samples of the bacterial culture medium were taken at two time points: 1 h and 24 h. Cellular metabolites of *E. coli* were extracted using previously optimized method (33). Samples were centrifuged at 3,220 × *g* at 4°C for 20 min. The supernatants were removed, and the bacterial pellets were washed twice with 1 mL cold normal saline. A 300 µL cold chloroform-methanol-water (1:3:1, vol/vol) extraction solvent containing 1 µM internal standards (CHAPS, CAPS, PIPES, and Tris) was used to dissolve the pellets. All of the samples were then frozen in liquid nitrogen, thawed on ice, and vortexed to liberate the intracellular metabolites. The samples were centrifuged for 10 min at 3,220 × *g* at 4°C after the third freeze-thaw cycle, and then, 300 µL of the supernatants was added to 1.5-mL Eppendorf tubes. The particles from samples were detached then centrifuged at 14,000 × *g* at 4°C for 10 min. A 200 µL supernatant was transferred into injection vials and stored at −80°C until analysis. Quality control samples were obtained by transferring 10 µL of each sample into a tube and mixing uniformly.

### Liquid chromatography-high-resolution mass spectrometry analysis

The metabolites were analyzed by a high-resolution mass spectrometry (LC-MS) equipped with a high-performance liquid chromatography system (RSLC U3000; Thermo Fisher, San Jose, CA); Q-Exactive Orbitrap mass spectrometer (Thermo Fisher, San Jose, CA) operated in both positive and negative electrospray ionization modes (rapid switching) at a 35,000 resolution with a detection range of *m/z* 50 to 1,000. The separation was performed on a HILIC column (2.1 × 100 mm, 1.8 µm; Waters, Ireland) coupled with guard column operated at 40°C. The mobile phase consisted of 10 mM ammonium acetate in water (solvent A) and acetonitrile (solvent B) running at the flow rate of 0.3  mL/min. The gradient elution mode started from 20% B to 50% B over 15 min, then to 95% B at 18 min, followed by a wash with 95% B for 3 min, and re-equilibration for 8 min with 20% B. The analysis injection volume was 10 µL, and the run time was 32 min. All samples were analyzed as a single LC-MS batch to avoid batch-to-batch variation.

### Data processing and bioinformatic and statistical analyses

The resulting raw data file was processed by the Progenesis QI program (Waters). Data were imported, processed, and aligned; peak selection was performed at a minimum intensity of 50,000 followed by subsequent metabolite identification. Presumptive metabolites were identified by LC retention time and *m/z* value. The maximum retention time shift for peak alignment was set as 0.2 min, and the mass tolerance was set as 5 ppm. Metabolites were then normalized by the median, log_2_ transformed, and scaled; statistical data analysis was carried out using the MetaboAnalyst 5.0 web portal. A PLS-DA was performed for all groups at each time points. One-way analysis of variance was used to determine significantly changed metabolites (*P*-value <0.05) and to identify false discovery rate (FDR) < 0.05 and fold change (FC) ≥2 (log_2_FC ≥1 or ≤−1). The FC is calculated as the ratio between the averages of the replicates from the treatment group and the control group at the same sampling timepoint. Pathway analysis was performed using Kyoto Encyclopedia of Genes and Genomes database. The histogram and heatmap were plotted with GraphPad Prism (8.0) and R (v 4.1), respectively.
